# Supplementary material for: SARS-CoV-2-specific T-cell epitope repertoire in convalescent and mRNA-vaccinated individuals
Source: Nat Microbiol. 2022 Apr 28;7(5):675–9. doi: 10.1038/s41564-022-01106-y (PMC9064790; doi:10.1038/s41564-022-01106-y)
Supplement: Supplementary file 1 — Reporting Summary [file 41564_2022_1106_MOESM1_ESM.pdf]

nature research

Corresponding author(s): Robert Thimme
Maike Hofmann
Christoph Neumann-Haefelin

Last updated by author(s): Mar 10, 2022

Reporting Summary

Nature Research wishes to improve the reproducibility of the work that we publish. This form provides structure for consistency and transparency in reporting. For further information on Nature Research policies, see our Editorial Policies and the Editorial Policy Checklist.

Statistics

For all statistical analyses, confirm that the following items are present in the figure legend, table legend, main text, or Methods section.

n/a	Confirmed	
☐	☒	The exact sample size (*n*) for each experimental group/condition, given as a discrete number and unit of measurement
☐	☒	A statement on whether measurements were taken from distinct samples or whether the same sample was measured repeatedly
☐	☒	The statistical test(s) used AND whether they are one- or two-sided<br>*Only common tests should be described solely by name; describe more complex techniques in the Methods section.*
☐	☒	A description of all covariates tested
☐	☒	A description of any assumptions or corrections, such as tests of normality and adjustment for multiple comparisons
☐	☒	A full description of the statistical parameters including central tendency (e.g. means) or other basic estimates (e.g. regression coefficient) AND variation (e.g. standard deviation) or associated estimates of uncertainty (e.g. confidence intervals)
☐	☒	For null hypothesis testing, the test statistic (e.g. *F*, *t*, *r*) with confidence intervals, effect sizes, degrees of freedom and *P* value noted<br>*Give P values as exact values whenever suitable.*
☒	☐	For Bayesian analysis, information on the choice of priors and Markov chain Monte Carlo settings
☐	☒	For hierarchical and complex designs, identification of the appropriate level for tests and full reporting of outcomes
☒	☐	Estimates of effect sizes (e.g. Cohen's *d*, Pearson's *r*), indicating how they were calculated

Our web collection on statistics for biologists contains articles on many of the points above.

Software and code

Policy information about availability of computer code

Data collection	All software used to perform data collection are described in the methods section of the manuscript or the supportive information. Multiparametric Flow cytometry data was collected by FACSDiva software version 10.6.2 (BD, Germany). ELISA data was collected by SparkControl magellan software version 2.2.
Data analysis	Multiparametric Flow cytometry data was analyzed using FlowJo software version 10.6.2 (Treestar, Becton Dickinson). Visualization and statistical analysis was performed using GraphPad 8 software. Sequence homology analyses were performed in Geneious Prime 2020.0.3 (https://www.geneious.com/) using Clustal Omega 1.2.2 alignment with default settings.

For manuscripts utilizing custom algorithms or software that are central to the research but not yet described in published literature, software must be made available to editors and reviewers. We strongly encourage code deposition in a community repository (e.g. GitHub). See the Nature Research guidelines for submitting code & software for further information.

Data

Policy information about availability of data

All manuscripts must include a data availability statement. This statement should provide the following information, where applicable:

- Accession codes, unique identifiers, or web links for publicly available datasets
- A list of figures that have associated raw data
- A description of any restrictions on data availability

Sequences of tested epitopes, sequences of used spike overlapping peptides as well as a list summarizing all CD8+ and CD4+ T cell responses to overlapping peptides are available in the community repository "Open Science Framework" and can be found via https://osf.io/zbk6q/. Additional raw and analyzed data and materials include pseudonymized patient data that may be subject to confidentiality. Requests of this data are promptly reviewed by the University of Freiburg Center for Technology Transfer to verify if the request is subject to any intellectual property or confidentiality obligations. Patient-related data not included in the

paper were generated as part of clinical examination and may be subject to patient confidentiality. Any data and materials that can be shared will be released via a Material Transfer Agreement.

Materials and Correspondence:
Christoph Neumann-Haefelin: christoph.neumann-haefelin@uniklinik-freiburg.de
Maike Hofmann: maike.hofmann@uniklinik-freiburg.de
Robert Thimme: robert.thimme@uniklinik-freiburg.de
Hugstetter Straße 55, 79106 Freiburg, Germany

Field-specific reporting

Please select the one below that is the best fit for your research. If you are not sure, read the appropriate sections before making your selection.

☒ Life sciences ☐ Behavioural & social sciences ☐ Ecological, evolutionary & environmental sciences

For a reference copy of the document with all sections, see nature.com/documents/nr-reporting-summary-flat.pdf

Life sciences study design

All studies must disclose on these points even when the disclosure is negative.

Sample size	Patients were recruited and patient material was banked at the University Hospital Freiburg; inclusion criteria were: (1) 16 individuals that received a prime and boost vaccination with the mRNA vaccine bnt162b2/Comirnaty, (2) 19 acutely infected and convalescent individuals following a mild course of SARS-CoV-2 infection, SARS-CoV-2 infection was confirmed by positive PCR testing from oropharyngeal swab and/or SARS-CoV-2 spike IgG positive antibody testing. No sample size calculations were performed. 16 vaccinated health care workers gave informed consent and were available to donate blood samples. Therefore, similar numbers of COVID-19 convalescents were selected.
Data exclusions	No data was excluded from the analysis
Replication	Analyses were performed in independent experiments. Findings were reproducible. Flow cytometry analysis: 19 convalescent individuals following a mild course of SARS-CoV-2 infection were analyzed up to 7 months after infection (3 outliers up to 12 months). Moreover, 16 individuals were screened 2-4 weeks after first mRNA boost vaccination (Pfizer/BioNTech, bnt162b2) and 7 of the same individuals 2-4 weeks after second boost vaccination (Pfizer/BioNTech, bnt162b2) Three individuals were analyzed who had a mild course of SARS-CoV-2 infection and were vaccinated once with mRNA vaccine (Pfizer/BioNTech, bnt162b2).
Randomization	Vaccinated donors and donors with a history of natural SARS-CoV-2 infection were selected based on availability and HLA-typing. The covariates age and gender are well-documented: Median age of vaccinated donors was 36 years, donors with a history of natural SARS-CoV-2 infection was 36 years. The gender ratio of vaccinated donors was m/f: 10/6, donors with a history of natural SARS-CoV-2 infection was m/f: 11/8.
Blinding	Non-objective parameters were not included in the study design and standardized analyses were applied. Thus, blinding was not considered necessary since biased analysis can be excluded.

Reporting for specific materials, systems and methods

We require information from authors about some types of materials, experimental systems and methods used in many studies. Here, indicate whether each material, system or method listed is relevant to your study. If you are not sure if a list item applies to your research, read the appropriate section before selecting a response.

Materials & experimental systems

n/a	Involved in the study	
☐	☒	Antibodies
☒	☐	Eukaryotic cell lines
☒	☐	Palaeontology and archaeology
☒	☐	Animals and other organisms
☐	☒	Human research participants
☒	☐	Clinical data
☒	☐	Dual use research of concern

Methods

n/a	Involved in the study	
☒	☐	ChIP-seq
☐	☒	Flow cytometry
☒	☐	MRI-based neuroimaging

Antibodies

Antibodies used	Anti-CD8-APC (SK-1, 1:200), BD, Cat#345775<br>anti-CD4-efluor450 (RPA-T4, 1:250), eBioscience, Cat#48-0049<br>anti-IFN-γ-FITC (25723.11, 1:8), BD, Cat#340449<br>fixable Viability Dye (eFluor506 1:200, 1:400), eBioscience, Cat#65-0866

Validation	All antibodies were obtained from commercial cendors and we based specificity on descriptions and information provided in corresponding data sheets available and provided by the manufacturers. Standardized analysis in different cohorts, antibody titration on PBMCs including unstained controls, comparisons of different antibody clones and conjugates and validated by publications:<br>CD4: antibody titration on PBMCs; control clones SK3; using B cells as negative control<br>CD8: antibody titration on PBMCs; control clone GHI/75; using B cells as negative control<br>IFNy, clone 25723.11: antibody titration on PBMCs; control clone 4S.B3; validated with respect to differential expression of activated and non-activated T cell subpopulations<br>Viability Dye was titrated on PBMCs; validated with respect to differential staining of live and dead cell populations

Human research participants

Policy information about studies involving human research participants

Population characteristics	Median age of vaccinated donors (n=16) was 36 years, donors with a history of natural SARS-CoV-2 infection (n=19) was 36 years. The gender ratio of vaccinated donors was m/f: 10/6, donors with a history of natural SARS-CoV-2 infection was m/f: 11/8.
Recruitment	Vaccinated donors as well as SARS-CoV-2-infected and SARS-CoV-2-convalescent patients were recruited at the University Hospital Freiburg; self-selection bias or other biases can be excluded since several people were included in the recruitment. Samples were banked and retrospectively selected. Banked samples from sex-, age- and HLA-matched vaccinated and convalescent individuals were retrospectively selected.
Ethics oversight	Written informed consent was obtained from all participants and the study was conducted according to federal guidelines, local ethics committee regulations (Ethik-Kommission der Albert-Ludwigs-Universität Freiburg, Freiburg, Germany; vote #: 322/20, #21-1135 and 315/20) and the Declaration of Helsinki (1975).

Note that full information on the approval of the study protocol must also be provided in the manuscript.

Flow Cytometry

Plots

Confirm that:

☒ The axis labels state the marker and fluorochrome used (e.g. CD4-FITC).

☒ The axis scales are clearly visible. Include numbers along axes only for bottom left plot of group (a 'group' is an analysis of identical markers).

☒ All plots are contour plots with outliers or pseudocolor plots.

☒ A numerical value for number of cells or percentage (with statistics) is provided.

Methodology

Sample preparation	Cryopreserved isolated human PBMCs were thawed and prepared for flow cytometry or in vitro expansion described in the methods section
Instrument	FACSCanto II
Software	FlowJo_v10.6.2 (Treestar)
Cell population abundance	Abundance of SARS-CoV-2-specific T cells are low (<10^-4 %)
Gating strategy	Lymphocytes gated on FSC-A and SSC-A, Doublet exclusion on FSC-H and FSC-W, Exclusion of dead cells, Gating on CD8+ or CD4+ cells.

☒ Tick this box to confirm that a figure exemplifying the gating strategy is provided in the Supplementary Information.
